# The effects of opium on the cardiovascular system: a review of side effects, uses, and potential mechanisms

**DOI:** 10.1186/s13011-020-00272-8

**Published:** 2020-04-17

**Authors:** Samaneh Nakhaee, Saeedeh Ghasemi, Kimiya Karimzadeh, Nasim Zamani, Samaneh Alinejad-Mofrad, Omid Mehrpour

**Affiliations:** 1 Medical Toxicology and Drug Abuse Research Center (MTDRC), Birjand University of Medical Sciences, Birjand, Iran; 2 Student Research Committee, Birjand University of Medical Sciences, Birjand, Iran; 3 Department of Clinical Toxicology, Loghman-Hakim Hospital, Shahid Beheshti University of Medical Sciences, Tehran, Iran; 4 Faculty of nursing and midwifery, Birjand University of Medical Sciences, Birjand, Iran; 5 Rocky Mountain Poison and Drug Safety, Denver Health and Hospital Authority, 1391 Speer Blvd, 777 Bannock St. MC 0180, Denver, CO 80204 USA

**Keywords:** Opium, Cardiovascular diseases, Coronary artery disease

## Abstract

**Background:**

In Iran, as in many other Asian and Middle Eastern countries, some believe that opium has beneficial effects on cardiovascular system. Dependent patients suppose that opium has positive effects on cardiovascular function and can prevent or improve cardiovascular diseases; however, only few comprehensive studies evaluating such effects have been performed.

**Objectives:**

In this study, we sought to clarify the effect of opium on cardiovascular problems by incorporating the previous findings and the current information on the issue and to explain the possible mechanisms of this effect.

**Methods:**

The available human studies published up to October 30, 2019, were searched in different databases. Case-control, cohort, and cross-sectional studies were retrieved. Papers published in English or those with an English abstract were included. The risk of bias for each included study was assessed based on the Newcastle-Ottawa Scale (NOS). We then categorized the effects of opium on cardiovascular problems along with its probable underlying mechanisms of action.

**Results:**

In this study, most of the published articles suggested the adverse effects of opium on the cardiovascular system, including atherosclerosis, myocardial infarction, arrhythmia, low ejection fraction, and cardiovascular mortality; however, some articles reported the beneficial or impartial effects of opium on the cardiovascular system. In this article, we have categorized all the effects of opium on cardiovascular system; also, the proposed mechanisms of action of opium in each of the above-mentioned disorders are summarized.

**Conclusion:**

Although the available evidences were incoherent, it was mostly suggested that opium use does not protect against or improve cardiovascular problems.

## Background

Opium is a crude material retrieved from *Papaver somniferum,* which is quite effective in the treatment of acute and chronic pain but can also result in opium use disorder [1]. Opium use is a social and health problem in many countries, including Iran [2, 3], and it has been reported to be the most commonly used substance in this country [4, 5]. Opium mostly affects the central nervous system (CNS), but it can also affect the respiratory and cardiovascular systems [6–8].

In many Asian and Middle Eastern countries, including Iran, some believe that opium has beneficial effects on cardiovascular diseases (CVDs) although only few studies have been performed to confirm or reject this assumption [1]. Studies show that patients with chronic diseases, including diabetes, hypertension, and ischemic heart disease (IHD), use opium more than the general population because they assume it is efficient in treating or preventing cardiovascular disorders. This is even a common belief among the medical community [9].

Therefore, we have tackled an increasing trend toward opium use in our society and the results obtained from the very limited studies on the issue are contradictory. The aim of this review article was, therefore, to clarify the effect of opium on cardiovascular problems by integrating the previous findings and the current information on the issue and to explain the possible mechanisms of this effect.

## Methods

### Search strategy

In this systematic review performed in accordance with the instructions of Preferred Reporting Items for Systematic Reviews and Meta-Analyses (PRISMA), several databases including PubMed, Web of Science, Scopus, Embase, Ovid, Google Scholar, and Persian databases such as Magiran and SID were searched. We used MeSH headings, free-text terms, and combination of relevant keywords including “opium”, “papaver”, “opiate”, “opioid”, “coronary heart disease”, “coronary artery disease”, “myocardial infarction”, “cardiovascular”, “atherosclerosis”*,* “atherogenesis”, and “ischemic heart disease”. PICO frame of this review was defined as P: Opium user, C: Non-opium user, O: Cardiovascular Diseases risk.

For example, the search strategy in PubMed central using MeSH terms and free-text terms were as follow:

(((“Opium”[MeSH]) OR “papaver”[MeSH])) AND ((((((((“cardiovascular diseases”[MeSH]) OR “Coronary Artery Diseases”[Mesh]) OR “myocardial infarction”[Mesh]) OR “Atherosclerosis”[MeSH]) OR “Myocardial Ischemia”[MeSH]) OR “coronary artery bypass surgery”[MeSH]) OR “percutaneous coronary intervention”[MeSH])

((((((((“cardiovascular disease”[Title/Abstract]) OR “Coronary Artery Disease”[Title/Abstract]) OR “myocardial infarction”[Title/Abstract]) OR “Atherosclerosis”[Title/Abstract]) OR “percutaneous coronary intervention”[Title/Abstract]) OR “Myocardial Ischemia”[Title/Abstract]) OR “coronary artery bypass surgery”[Title/Abstract])) AND (((opium [Title/Abstract]) OR papaver [Title/Abstract]) OR opioid [Title/Abstract]). Two researchers independently searched the above-mentioned databases until October 30, 2019.

### Inclusion criteria and study selection

**Fig. 1:**
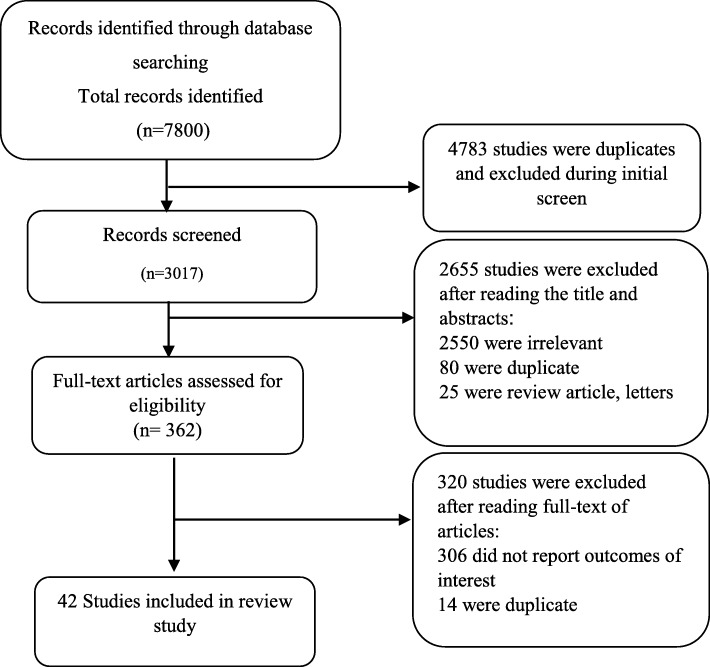
Flowchart of the literature search and strategy for the selection of the relevant documents

Case-control, cohort, and cross-sectional studies were retrieved. In order to find further articles on the subject, reference lists of the identified articles were also scanned. No restrictions were imposed on the language and study period. Papers published in English or those with an English abstract were included. Studies about natural opioids such as morphine and synthetic opioids such as methadone were not included in our review. Also, articles with inadequate data and no clear description of the methods, editorials, conference papers, and review articles were excluded. Irrelevant studies were excluded from the list by the assessment of their titles, abstracts, and full texts. The screening was performed after exclusion of duplicates. The primary outcome was defined as the risk of atherosclerosis, myocardial infarction, arrhythmia, effects on the ejection fraction index, the severity of CAD, and mortality from CADs (Table 1). We included studies assessing at least one of these primary outcome measures. The flowchart of our searching process for the selection of the included articles is presented in Fig. 1.

**Table 1:** Opium and Cardiovascular problems

Opium and	Atherosclerosis	Potential risk factor	[10–18]
		Potential protective factor	[1], [19]
		Impartial factor	[20–25]
	MI	Potential risk factor	[26–31]
		Potential protective factor	[32–35]
		Impartial factor	[36–40]
	Arrhythmias	Potential risk factor	[41], [37], [21], [39], [42], [43], [44], [45]
		Potential protective factor	[46]
		Impartial factor	[47]
	Ejection Fraction	Potential risk factor	[24], [22], [21, 48], [42], [49], [50]
		Impartial factor	[1], [12], [26], [32, 47], [16], [3, 51], [38], [33], [23, 39, 52–54]
	Cardiovascular mortality	Potential risk factor	[21], [24], [37], [39], [55]
		Potential protective factor	[32]
		Impartial factor	[3], [33], [26], [38], [53], [56], [6, 29, 47, 52, 57, 58]

### Data extraction

**Fig. 2:**
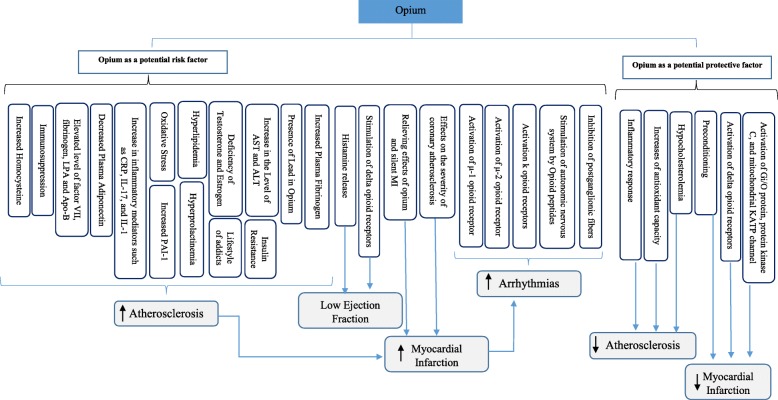
Potential mechanism for effects of opium on cardiovascular problems**.**: Increased risk of different complications**.**: Decreased risk of different complications

Data were extracted using a pre-defined checklist and were assessed by two independent reviewers. The data consisted of the date and place of the study, type of study, sample size and sampling method, information about the participants, the definition of exposure and outcome, route and amount of exposure, age, and gender of the participants, methods of assessment, confounding variables, and the main findings of each study with principal measures (e.g., risk ratio, difference in means, etc.). The risk of bias for each included study was assessed based on the Newcastle-Ottawa Scale (NOS). Studies with a score of 5 or more were considered as high-quality. None of the articles meeting our inclusion criteria were excluded. Finally, we tried to categorize the effects of opium on cardiovascular problems along with identifying its probable underlying mechanisms of action (Fig. 2).

## Results

### Opium and atherosclerosis

Some previous studies mention that opium is a risk factor for atherosclerosis, while others advocate its positive effects. There are also a few studies ascribing that opium does not affect atherosclerosis.

### Opium as a potential risk factor for atherosclerosis

A study of 2405 patients showed that using opium was a risk factor for CADs (OR:1.8, CI: 1.1–3.1, *P* = 0.015). This study also excluded cigarette smokers from the analysis and obtained the same general findings. The amount of the consumed opium was found to be significantly associated with the severity of atherosclerosis (r = 0.2, *P* = 0.002) [10]. In another study, the risk factors for CADs were evaluated in young men and women undergoing angiography. Patients were categorized based on CADs, and the results showed that CADs were related to diabetes and increased LDL and lipoprotein A in women and cigarette smoking and opium use in men. In all the patients, opium use was the second significant factor affecting CADs following diabetes (OR:2.65, CI:1.31–5.35, *P* = 0.007) [11].

The results of a cross-sectional study of 1925 diabetic patients undergoing angiography (228 opium users and 1697 non-users) showed that CADs were more severe in opium dependent patients. The average Gensini score and the extent score used to assess the severity of CADs were significantly higher in those with opium use disorder. There was no relationship between the route of opium use (inhaled or orally consumed) and the severity of CADs, but the consumed dose of opium was related to this variable [12].

Darabad et al. investigated 1170 patients undergoing coronary artery angiography (121 and 1049 with and without a history of opium use, respectively). The researchers concluded that older age (OR:0.68,P:0.00), diabetes (OR:2.416, P:0.00), hypertension (OR:2.007, P:0.000) and opium use (OR:2.083, P:0.019) were the risk factors for CADs although no difference was detected in the number of the involved coronary arteries [13]. In another study, 242 patients undergoing angiography were investigated. The relative frequency of stenosis ≥50% and the number of the involved arteries were significantly higher in dependent patients. Multiple logistic models of age, gender, and opium use demonstrated a significant relationship between opium use and CADs [14].

Nadimi et al. evaluated 250 patients with evidence of stable angina, heart ischemia in a cardiac stress test, and class I or II symptoms in coronary angiography. The patients were examined in two 125-patient groups; one with microvascular coronary dysfunction (MCD) and the other with normal angiography and no MCD. The two groups were homogeneous regarding gender, age, history of diabetes, blood pressure, and blood lipids. Based on the results, opium use disorder was reported to be a prognostic factor for MCD (OR = 3.575, CI 95%: 1.161–9.016, *P* = 0.0069) [15].

Another study was performed to determine the association between acute opium use and CADs in 91 candidates for angiography. Those with positive results in catheterization were selected as cases and those with normal angiography were assigned to the control group. The two groups were similar in demographic information and risk factors. The results showed that opium use disorder was a risk factor for CADs (OR: 3.8, CI 95%:1.5–9.5). In cases, the number of involved arteries and the degree of coagulation in them were not significantly different between dependent and non-dependent patients [16]. The results of an experimental study revealed a surge in the levels of triglycerides (TG), cholesterol, LDL, and atherogenic index of plasma in non-addicted and addicted hypercholesterolemic rabbits as time passed, with a significantly more severe deterioration in addicted hypercholesterolemic rabbits. Arteriosclerotic plaques of the aorta were significantly more in the addicted hypercholesterolemic rabbits.. It was concluded that oral consumption of opium had aggravating effects on the formation of arteriosclerotic plaques and exacerbated the atherogenic effects of high-fat foods [17]. Another study evaluating postmortem heart diseases in 168 addicted bodies showed that of the 35 cases with CADs, 28 had significant stenosis in one to more than four coronary arteries due to arteriosclerotic plaques [18].

#### Potential mechanisms

##### Inflammation

Atherosclerosis depends on the immune response [59]. Some studies on substance use disorder consider opium as an immunosuppressive factor [59] and reveal an increase in inflammatory mediators such as C-reactive protein (CRP), interleukin-17, and interleukin-1 receptor antagonist in opium users. Thus, aggravation of inflammation increases atheroma formation in dependent patients [40]. In a study of 15 dependent and 15 non-dependent patients with involved coronary arteries and an ejection fraction of more than 35%, the plasma levels of interleukin-6 and interleukin-1 receptor antagonists were evaluated. The level of interleukin-1 receptor antagonist was higher in the dependent cases, but opium did not have a significant effect on the plasma level of interleukin-6 [59]. Thirty-five dependent smokers were compared to 35 non-dependent smokers in another study, which revealed that the level of factor VII was significantly higher in addicted cases [60]. One study showed that the levels of CRP, factor VII, fibrinogen, lysophosphatidic acid (LPA), and apolipoprotein B (Apo-B) were higher in patients with opium use disorder, while the levels of LDH and Apo-A were reported to be significantly lower in them compared to non-users [40].

##### Hyperlipidemia

Opium consumption may have undesirable effects on lipid metabolism [61, 62]. Some clinical studies have shown that opium may affect the levels of total cholesterol, TG, LDL cholesterol, and HDL cholesterol [17, 32, 63–65].

Increase in the levels of aspartate (AST) and alanine aminotransferase (ALT) substance use may increase the levels of AST and ALT, which in turn, can cause severe atherosclerosis in the coronary arteries [3].

##### Oxidative stress

Oxidative stress plays a critical role in the pathogenesis of atherosclerosis. It has been advocated that morphine and heroin decrease the total antioxidant capacity irrespective of cigarette smoking. Therefore, elevated oxidative stress may involve in atherosclerosis in opium users [40].

##### Increased Homocysteine

Increased homocysteine is one of the factors that can result in the advancement of atherosclerosis and the development of CADs. It has been demonstrated that opium use disorder is strongly related to increased homocysteine, and consequently, the elevated risk of developing CADs [66].

##### Increased plasma fibrinogen

Increased plasma fibrinogen is also considered as an independent risk factor for CADs and can increase the risk of thrombosis. Some studies have reported that plasma fibrinogen is significantly higher in opium-addicted patients [67–69].

##### Increased plasminogen activator Inhibitor-1 (PAI-1)

PAI-1 inhibits the formation of plasmin through inhibitory effects on plasminogen activator and consequently prevents the clotting cascade in the arteries. A study conducted among 160 patients with CADs showed that the average serum level of PAI-1 was higher in addicted patients with congestive heart disease (CHD) [70].

##### Decreased plasma Adiponectin

Adiponectin has anti-diabetic and anti-atherogenic effects, and when decreased, it promotes the risk of developing metabolic disorders such as insulin resistance and cardiovascular diseases (CVDs) in patients with opium use disorder. The relationship between adipokinase and opium use disorder can indirectly play a role in the development of atherosclerosis. One case-control study demonstrated that the serum level of adiponectin was significantly lower in patients with opium use disorder [71].

##### Deficiency of testosterone and estrogen

In men, low testosterone level can cause CADs and increase the risk of cardiovascular-related mortality. The level of plasma testosterone is lower in those with opium use disorder. Similarly, low level of estrogen and reduction of estrogen in the pre-menopause period have been reported in addicted females, which can cause CADs [40].

##### Hyperprolactinemia

Opium increases the level of prolactin in the blood. Prolactin can induce smooth muscle cells growth through a mechanism that depends on protein kinase C and may also play a role in the hyperplasia of muscle cells of the arteries and lead to atherosclerosis [40].

##### Insulin resistance

Similar to type 2 diabetic patients, people with opium use disorder show insulin resistance and an increase in the insulin level of the blood. Insulin resistance results in the atherosclerosis of coronary arteries and the advancement of coronary plaques [40].

##### Presence of Lead in opium

The presence of impurities such as lead in opium can be a factor causing adverse effects on the cardiovascular system. Lead has been reported to be present in opium samples, and blood lead level has been shown to be high in those with opium use disorder [72–74]. Some studies have indicated a relationship between blood lead concentration, hypertension, and development of atherosclerosis [75].

##### Lifestyle

Opium is a CNS suppressant and can decrease physical activity in addicted individuals, which increases the risk of CVDs [40]. In a study, depression, reduction of physical activity, and obesity were all reported in addicted patients [76]. Also, some studies showed that opium users followed treatment and nutrition advice and performed cardiovascular activities less than others [3, 51].

### Opium as a potential protective factor

One study investigating the effects of long-term use of opioids on the extent of CADs showed that the coronary arteries clogged less severely in opioid dependents [19]. In a cross-sectional study conducted on 325 patients who were candidates for coronary artery bypass grafting ([CABG], 117 opium users and 208 non-users), fewer coronary arteries were involved in opium users, and there was no relationship between the number of involved coronary arteries and stenosis and long-term opium use [1].

### Potential mechanism

Some studies reflect that opioids can reduce inflammation and CADs [19, 40]. It has been stated that long-term opium use reduces atherosclerosis directly and protects against ischemic and infarction damages [19]. As there are more than 40 different alkaloids in opium, more research is required to better understand the effect of opium on the inflammatory response. Several other studies have shown that opium increases serum antioxidant capacity [77]. Gülçin et al. also reported a robust antioxidant capacity for morphine in vitro [78]. Several studies have demonstrated a remarkable hypocholesterolemia pattern in patients with substance use [79]. All of the issues mentioned above mandate the necessity of more in-depth research on this subject.

### Opium as an impartial factor

Some studies deem the effects of opium on the development of atherosclerosis to be insignificant. A study on 299 candidates for coronary angiography showed that opium users had a higher chance of acute CADs, although this relationship was not statistically significant [20]. In a study of 268 patients with CADs, it was revealed that smoking and recent myocardial infarction (MI) were more common in patients with opium use disorder, and hyperlipidemia and diabetes were more common in patients without opium use disorder. The results showed that there was no statistically significant difference in the number of involved coronary arteries between the two groups of people with and without opium use disorder [21].

The results of another study on 1339 patients who were candidates for coronary artery bypass showed that the number of involved coronary arteries, more than 50% stenosis of the main left artery, and the extent of carotid stenosis were not different between patients with and without opium use disorder [6]. A study on 232 CAD patients showed that the number of involved coronary arteries was similar between these two groups [22]. Rezvani (2012), in a cross-sectional study on 81 patients suffering from IHD, showed that orally consumed (OR: 0.91, CI 95%: 0.508–1.65, P: 0.88) or inhaled opium (OR:1.4, CI95%: 0.65–3.02, P: 0.39) did not significantly affect IHD. Oral consumption of opium was a protective factor against stroke but not against IHD [23]. In a study of 566 patients undergoing CABG, it was demonstrated that patients with a positive history of opium use (82 people) were similar to non-users (484 people) regarding the number of the involved arteries [24].

Shahryari conducted an experimental study on male hamsters to investigate the influence of daily opium use on lipid profile and aortic atherosclerosis in one month. The results showed that the levels of LDL and TG were significantly higher, and HDL level was significantly lower in opium dependent hamsters than the control group. In addition, histopathologic changes in the heart (i.e., fatty streak, fibrous plaque, and calcification) were not different between the groups [25].

### Opium and MI

Myocardial infarction occurs due to a block in the blood flow to a part of the myocardium. There are different risk factors for this condition, and some believe that endorphin and endogenous opioids play an important role in the development of MI, while others point to their protective or ineffective role in the development of MI.

### Opium as a potential risk factor

Roohafza et al. investigated 469 patients suffering from acute MI in a cross-sectional study and concluded that opium use disorder reduced the age of MI up to 3.6 years (*P* = 0.003, CI 95%:1.2–6.0) [26]. In a case-control study, 118 MI patients in the coronary care unit were compared with 118 heart-healthy patients admitted to other wards. The results showed that opium use had a relationship with MI (OR = 26.3, 95% CI:7.5–92.4, *P* < 0.01), but the type of MI and its extent were not statistically different between patients with and without opium use disorder in the group suffering from MI [27].

Another nested case-control study conducted among 11,693 patients suffering from MI showed that using opioids increased the risk of MI up to 1.28 times (95% CI: 1.19–1.37) and this risk was higher in patients receiving morphine (OR: 1.71, CI 95%: 1.09–2.68), meperidine (OR: 2.15, CI 95%: 1.24–3.74), and mixed treatments (OR: 1.46, CI 95%: 1.22–1.76) [28]. Bartolucci et al. performed a study on 18,048 MI patients, 285 of whom were opium users. They showed that users had a significantly higher risk of ST-elevation and infarction in the anterior wall [29]. Another study demonstrated that creatinine phosphokinase, lactate dehydrogenase, and CKmb enzymes were higher in patients with opium use disorder and acute MI [30].

An experimental study evaluated the effects of inhaled opium on rabbits and showed that using opium increased the level of troponin-I and exacerbated electrocardiographic (ECG) changes. It was also revealed that long-term use of opium raised the frequency of ST elevation. Exposure to opium reduced myocardium degeneration after the induction of ischemia but increased tissue congestion and hemorrhage [31].

### Potential mechanism

The high prevalence of ischemic changes in ECG (ST-T changes in the ECG) and increased heart enzymes in patients with opium use disorder can be related to the relieving effects of opium on chest pain and the high prevalence of silent MI in these patients [1]. Also, the relationship between opium use and MI can be a result of opium effects on the development of coronary atherosclerosis.

### Opium as a potential protective factor

A study among 460 patients suffering from acute MI (239 people with opium use disorder and 221 without opium use disorder) revealed that the emergence of MI in the anterior wall was significantly less common in opium users. The emergence of posterior wall infarction was similar between the two groups [32]. The results of a retrospective study investigating the information of 1545 men undergoing percutaneous coronary intervention (PCI) from the data bank of Tehran Heart Center showed that the ratio of involvement of the left anterior descending artery was higher in non-users. However, the reference vessel diameter and lesion length were not different between the two groups [33]. Another experimental study on the rabbit heart showed that morphine reduced cardiomyocyte apoptosis through the activation of delta-opioid receptors [34]. Research on the mouse heart showed that kappa opioid receptors in the heart caused enlargement in the infarction area, while delta-opioid receptors had protective effects [35].

### Potential mechanism

Activation of delta-opioid receptors prior to ischemia reduced the infarct area in several animal models, including rats [80], rabbits [81], and pigs [82]. Recent data has also shown that opioid peptides are involved in a phenomenon called preconditioning, which protects the heart from the damages of long-term ischemia by temporary ischemia or hypoxia and reduces the size of the infarct area. Shultz et al. mentioned that the induction of preconditioning through morphine and delta-1 opioid receptor agonists proved the role of opioid peptides in the preconditioning and cardioprotective effect [83, 84]. It has also been demonstrated that delta receptor-selective agonists enforce protective effects in the heart myocytes through the activation of Gi/O protein, protein kinase C, and finally, the mitochondrial KATP channel [85].

### Opium as an impartial factor

A case-control study conducted among 150 MI patients hospitalized in the intensive care unit (ICU) and 150 patients hospitalized in the surgery ward showed that opium use disorder was not significantly different between the cases and controls (OR = 1.71, *P* > 0.05) [36]. Also, the results of another study on 116 MI patients showed that the number of extensive infarctions was twice in people with opium use disorder, but this difference was statistically insignificant [37]. Another study on 160 males diagnosed with acute MI also confirmed that there were no differences between those with and without opium use disorder in terms of location of the MI, angiography findings, and the level of troponin-I [38]. Rostamzadeh and Khademvatani reported that there was no statistically significant difference between the two groups of opium users and non-users regarding the prevalence of ST-elevated MI (STEMI), location of MI, and the peak amount of CPK-MB [39]. Overall, the majority of the studies pinpoint opium as a risk factor for MI. This substance can hide MI symptoms through its pain-relieving properties,and consequently, delay treatment and cause lesion progression [40].

### Opium and arrhythmias

#### Opium as a potential risk factor

It has been previously mentioned that opium use disorder is a risk factor for the incidence of arrhythmias after acute MI irrespective of the patients’ age, gender, ejection fraction, and the extent of infarction (*P* = 0.001; CI: 7.26–28.65; OR = 14.60). Premature ventricular contractions (PVCs) and ventricular tachycardia (VT) are significantly more common in opium-addicted individuals [41]. The results of a cross-sectional study on 116 patients suffering from MI revealed that ventricular arrhythmias such as PVCs, VT, and ventricular fibrillation (VF) were significantly more prevalent in patients with opium use disorder [37]. Another study on 268 addicted and non-addicted CAD patients showed that complications after cardiac arrhythmia surgery were significantly higher in opium users [21]. Also, it was proved that VT and VF were more common in dependent patients following MI [39]. In patients with opium and ephedrone use, sinus tachycardia, ventricular extrasystole, and supraventricular extrasystole were diagnosed in 4.67, 7.4, and 6.11% of patients, respectively [42]. Another cross-sectional study showed that 60.6 and 54.6% of patients with opium and methadone toxicity had QT prolongation, respectively [86].

The combined effect of opium use and hypercholesterolemia was investigated on fatal heart arrhythmia in an experimental study on rabbits, which showed that long-term use of opium increased the atherogenic index of plasma. For a short period, QT interval increased significantly in the hypercholesterolemic group, but the index was similar in the two groups in the long run [43]. The results of another study showed that morphine and pentazocine had arrhythmogenic effects on the pig heart [44]. A case report demonstrated atrial flutter in an infant whose mother was addicted to cocaine and opiates. The infant was born to a 35-year-old mother through cesarean section (due to fetal tachycardia) in the 36th week of gestation and weighted 3.1 kg. It had atrioventricular tachycardia, and ultimately, an atrial flutter developed [45].

#### Potential mechanisms

The initial pathophysiology of arrhythmia is related to dysfunction in the conduction system. Opioid receptors in the ventricles and atria may play an essential role in the development of different arrhythmias [87, 88] by increasing heart rate and causing changes in heart rhythm [89, 90]. It has also been claimed that the μ-1 opioid receptor causes tachycardia, and μ-2 causes bradycardia [91]. Based on evidence, kappa opioid receptors can be involved in the development of an arrhythmic response. Coles et al. showed that the activation of kappa opioid receptors in pigs caused arrhythmia in them. They demonstrated that neither pentazocine nor morphine had protective effects on the heart, and the pro-arrhythmic effects of these medications were removed through blockage of the k receptors [44].

Several studies suggest that the potential arrhythmogenic activities of opioids at small doses occur through the activation of kappa opioid receptors, and their anti-arrhythmic actions at larger doses occur due to direct interaction with heart cell membranes [92]. Opioid peptides have also been advocated to stimulate the autonomic nervous system and raise heart rate and systolic blood pressure, which may cause arrhythmia per se [87]. Endogenous opioids, together with the sympathetic and parasympathetic systems in the brain stem, control the cardiovascular function. The postganglionic fibers innervating the rabbit heart have been shown to contain opioid and cannabinoid receptors, through the inhibition of which, neurogenic tachycardia ensues [93].

#### Opium as a potential protective factor

In a cross-sectional study on the patients undergoing CABG, it was revealed that using opium was one of the factors that significantly prognosticated atrial fibrillation after CABG (OR = 0.37, 95% CI: 0.93–1; *P* = 0.02) [46].

#### Opium as an impartial factor

A cross-sectional study on 304 patients with acute MI showed that patients with and without opium use disorder did not have any difference in the development of arrhythmia [47].

### Opium and ejection fraction (EF)

#### Opium as a potential risk factor for decreasing EF

It was previously shown that patients with a history of opium use had a significantly lower EF compared to those who did not use opium, although the functional class was similar between the two groups [24]. A study of 232 diabetic CAD patients showed that EF was significantly lower in opium users with a statistically similar functional class [22]. In addition, two other studies conducted on patients undergoing CABG showed that EF was significantly lower in opium users [21, 48].

In 1993, 65 patients who used opium and ephedrone were investigated by 24-h Holter monitor, ECG, and echocardiography. Although none of the patients had heart failure and cardiac muscle contractions were normal, echocardiography showed abnormal changes in the left ventricle including ventricular enlargement, reduced EF, and systolic shortening of myocardial fibers which were indicators of a decline in the compensatory mechanisms of the myocardium in substance users [42]. In another study conducted on patients undergoing coronary artery grafting surgery, 50 opium users and 50 non-users were compared regarding EF before the surgery. The results showed that average EF was lower in the opium users, but this difference was not statistically significant (*P* = 0.236). When the patients were categorized into the two subgroups of those with normal and abnormal EF, it was revealed that 56% of the patients with opium use disorder had abnormal EF (versus only 36% abnormal EF in non-opium users) before the surgery showing a significant difference. Left ventricular end-diastolic pressure was also higher in addicted patients [49].

Garg reported a case of poisoning due to the consumption of raw opium resulting in severe depression in a 13-year-old female who did not have any relevant medical history. On presentation, the patient was breathing shallowly, and the heart rate was over 150 bpm accompanied by hypotension (60/40 mmHg). Two-dimensional echocardiography showed left ventricular hypokinesia together with an LVEF of 10%. Heart enzymes had also increased (CK-MB = 34.71 ng/mL [normal range; 1.39–6.22 ng/mL] and troponin I:0.32 ng/mL [normal range: ≤ 0.014 ng/mL]). The patient recovered eventually, and EF increased to 56% [50].

#### Potential mechanism

Opium contains 80 different alkaloids, such as morphine and codeine. Morphine is a μ receptor agonist responsible for cardiovascular complications, including histamine release, and results in bradycardia, vasodilation, hypotension, and decreased cardiac output [94]. It has also been demonstrated that increased contractile response due to the stimulation of rat cardiac beta-adrenergic receptors is reversed through delta receptor agonism [95].

#### Opium as an impartial factor

A study on two groups of 228 diabetic patients undergoing angiography showed that EF was similar between opium users and non-users [12]. Roohafza et al. claimed that EF was similar between 126 opium users and non-users with acute MI on presentation and within one year after that [26]. EF and Killip class were similar between addicted and non-addicted patients in studies on 460 and 304 MI patients [32, 47]. Another study evaluating 53 patients with CAD and 33 patients with normal angiography concluded that EF was similar between patients with and without opium use disorder [16].

The results of two other descriptive studies on 782 and 200 male patients undergoing CABG also showed that the functional class and EF before the surgery and six months after that were similar between case and control groups [3, 51]. Evaluations performed on 325 CABG candidates (117 opium users and 208 non-users) showed that there was no relationship between the duration of opium use and EF before and after surgery [1]. Davoodi et al. also confirmed the results mentioned above [38]. Sharafi et al. retrospectively investigated 1545 male patients undergoing PCI (including 350 opium users) using Tehran Heart Center data bank and showed that addicted and non-addicted patients were similar regarding the extent of the lesion and EF [33]. Similar results were withdrawn from other studies, all confirming that EF, the emergence of heart failure after MI, and Killip class were similar between opium dependent and non-dependent patients [23, 39, 52–54].

### Opium and mortality in patients with CAD

#### Opium as a potential risk factor

A cohort study of 566 CABG patients followed the patients for 6.5 years to obtain their survival rate, which was reported to be 86.6% in opium users and 92.7% in non-users (Hasard Ratio:2.16; CI 95%: 0.96–4.84; P:0.06) [24]. The rate of mortality, angina after MI, cardiogenic shock, and cardiac arrest were shown to be significantly higher in opium users in another study [37], a result reported by other studies in this area, as well [39]. Najafi et al. showed that based on the EuroSCORE criteria, which is a method of predicting the possibility of mortality in patients undergoing heart surgery, the average score obtained from addicted patients was significantly higher than non-addicts [21]. In a cohort study conducted among 50,045 patients, opium use was suggested as a risk factor for mortality in these patients, even those who had used trivial amounts of opium. The use of opium significantly increased the overall risk of mortality (HR = 1.86; 95% CI: 1.68–2.06) and the mortality due to different causes such as circulatory diseases (HR = 1.81) and cancer (HR = 1.61). Among the deaths due to blood circulation, opium use increased the risk of IHD (HR = 1.9; 95% CI: 1.57–2.29) and cerebrovascular events [55].

#### Opium as a potential protective factor

A study of 460 MI patients revealed that inpatient mortality rate was lower in those with opium use disorder. Although this difference was not significant, in those with anterior wall infarction, the mortality was significantly higher in non-users [32].

#### Opium as an impartial factor

A retrospective study evaluating one-year major adverse cardiac events (e.g., cardiac death, non-fatal MI, need for target vessel revascularization, and target lesion revascularization) in 1545 patients revealed no significant difference between patients with and without opium use disorder (*P* = 0.312) [33]. Roohafza et al. claimed that the initial death rate was similar between these two groups. Also, they mentioned that one-year mortality and morbidity rates were also similar between them [26]. Another study demonstrated that although hospitalization period was more prolonged in opium users, hospital mortality, requiring re-hospitalization or re-vascularization, and the six-month mortality rate were still the same between them [38]. In 334 ST-elevation myocardial infarction (STEMI) patients, hospital outcome, mortality rate six months after MI, chest pain, and pulmonary edema were not different between the two groups [53].

Harati et al. evaluated the relationship between opium use, mortality rate and cardiac arrest in 400 MI patients. They concluded there was no significant difference between addicted and non-addicted groups regarding the study outcomes, although opium users were more commonly hospitalized due to cardiac problems [56].

Short-term postoperative complications were evaluated in a study by Safaii et al., which reported that rates of complications after surgery, rehospitalization in the ICU, hospitalization period after the surgery, and hospital mortality were similar between the groups [3]. Other studies reported similar insignificant differences between the two groups of patients regarding hospital mortality rate, early postoperative complications, and duration of intubation and ICU admission [6, 29, 47, 52, 57, 58].

### Opium and response to treatment

Jazi et al. examined the response to thrombolytic treatment in opium users in comparison to non-users and showed the beneficial effects of opium on the outcomes of thrombolytic therapy. Better response to thrombolytic agents indicates a smaller infarct size. Q-wave and ST-resolution after streptokinase injection were 63.8 and 47.2% in users and non-users, respectively (*P* < 0.05) [96]. The effect of opium use disorder on resistance to aspirin was also investigated in 260 patients suffering from angina, considering the measurement of 11-dehydroxy thromboxane B2 level (UTXB2) in urine samples as the index for aspirin resistance. It was found that aspirin resistance was significantly higher in patients with opium use disorder [97].

## Discussion

Most of the included studies did not support the protective effects of opium against CADs. Hasandokht [9] conducted a systematic review suggesting that opium did not have protective effects against CADs. It was also reported that age at the onset of CADs and hospital mortality were not protected by opium use. In another review article, it was noted that opium did not improve cardiovascular diseases. Instead, it harmed glycemic control, blood pressure, and lipid profile and caused atherosclerosis [40]. In addition to the adverse effect of opium on atherosclerosis of coronary arteries, some studies also noted that opium could be a risk factor for carotid stenosis [6]. The data from a large prospective cohort study showed the increased risk of death from circulatory diseases associated with opium consumption [55]. Some probable confounding factors should be considered in interpreting the results and design of the study. a) Traditionally, most opium users smoked tobacco. Hence, cigarette smoking should be considered as a confounding factor that was not reported in some included studies, which limits the generalizability of results. b) The dose of opium and the length, and frequency of opium use are also the issues that researchers should take into account. c) Since the opium available in the illicit market is not a pure substance and contains impurities such as lead, specifically in Iran, this factor can cause a difference in the results of the studies conducted on opium in different places around the world. To determine the exact effects of opium use on CADs further studies are required in this area.

## Conclusion

Although the results are controversial, most of the studies mentioned above did not support the protective effects of opium against cardiovascular problems. Future studies are recommended to evaluate the effect of the route and amount of opium use on CADs and the interaction between opium and cigarette smoking. Unfortunately, misconceptions as to the positive effects of opium are widespread, and healthy people, as well as patients with heart diseases or diabetes, should be informed of the dangerous effects of opium use on cardiometabolic diseases. Also, is recommended to promote the knowledge of medical communities and staff on the potential health consequences of opium consumption. Future studies should aim to identify the appropriate strategies to treat substance use disorder for primary and secondary prevention of cardiovascular problems.

## Data Availability

Not Applicable.

## Abbreviations

CNS: Central nervous system
CVDs: Cardiovascular diseases
IHD: Ischemic heart disease
OR: Odds ratio
MESH: Medical Subject Headings
MCD: Microvascular coronary dysfunction
TG: Triglyceride
LDL: Low-density lipoprotein
CRP: C-reactive protein
LPA: Lysophosphatidic acid
Apo-B: Apolipoprotein B
LDH: High-density lipoprotein
ALT: Alanine aminotransferase
AST: Aspartate aminotransferase
PAI-1: Plasminogen Activator Inhibitor-1
CHD: Congestive heart disease
CABG: Coronary artery bypass grafting
MI: Myocardial infarction
CKmb: Creatine kinase-MB
ECG: Electrocardiography
PCI: Percutaneous coronary intervention
ICU: Intensive care unit
STEMI: ST-elevated myocardial infarction
PVCs: Premature ventricular contractions
VT: Ventricular tachycardia
VF: Ventricular fibrillation
EF: Ejection fraction
LVEF: Left ventricular ejection fraction
HR: Hazard ratio
UTXB2: Urinary 11-dehydroxy thromboxane B2 level
